# Cyclin D1 targets hexokinase 2 to control aerobic glycolysis in myeloma cells

**DOI:** 10.1038/s41389-020-00253-3

**Published:** 2020-07-24

**Authors:** M. Caillot, J. Bourgeais, H. Dakik, É. Costé, N. M. Mazure, É. Lelièvre, O. Coqueret, O. Hérault, F. Mazurier, B. Sola

**Affiliations:** 1 Normandie Univ, INSERM, UNICAEN, Caen, France; 2 Service d’Hématologie Biologique, CHRU de Tours, Tours, France; 3 Université de Tours, GICC EA7501, CNRS, ERL7001 Tours, France; 4 Université Côte d’Azur, INSERM, Centre Méditerranéen de Médecine Moléculaire, Nice, France; 5 Paul Papin ICO Cancer Center, CRCINA, INSERM, Université de Nantes, Université d’Angers, Angers, France

**Keywords:** Haematological cancer, Cell biology

## Abstract

Cancer cells are characterized by the Warburg effect, a shift from mitochondrial respiration to oxidative glycolysis. We report here the crucial role of cyclin D1 in promoting this effect in a cyclin-dependent kinase (CDK)4/6-independent manner in multiple myeloma (MM) cells. We show that the cyclin D1 oncoprotein targets hexokinase 2 (HK2), a major glycolysis regulator, through two original molecular mechanisms in the cytoplasmic and nuclear compartments. In the cytoplasm, cyclin D1 binds HK2 at the outer mitochondrial membrane, and in the nucleus, it binds hypoxia-inducible factor-1α (HIF1α), which regulates *HK2* gene transcription. We also show that high levels of *HK2* expression are correlated with shorter event-free survival (EFS) and overall survival (OS) in MM patients. HK2 may therefore be considered as a possible target for antimyeloma therapy.

## Introduction

Multiple myeloma (MM) is a hematological malignancy characterized by the proliferation and accumulation of clonal plasma cells in the bone marrow. MM tumor cells are characterized by the overproduction of a monoclonal immunoglobulin (Ig) or a *λ*/*κ* light chain, causing hyperproteinemia, renal failure, bone lesions, and immunodeficiency. In recent years, new agents, such as immunomodulators, proteasome inhibitors, monoclonal antibodies, and checkpoint inhibitors, have been shown to be active against MM, but this disease remains incurable^1^. Cyclin D1 is expressed in a subtype of MM tumors, due to *t*(11;14)(q13;q32) translocation or biallelic dysregulation of the *CCND1* locus^2^. Consistent with the well-known role of cyclin D1 in regulating the cell cycle through cyclin-dependent kinase (CDK)4/6 activation and retinoblastoma protein (pRB) inactivation, the overexpression of cyclin D1 promotes the cell cycle, leading to uncontrolled proliferation^3^.

In addition to its role in cell-cycle regulation, cyclin D1 controls other mechanisms that are crucial for cell homeostasis, such as transcriptional regulation, genomic stability, senescence, and migration. These noncanonical functions may depend on the subcellular location of cyclin D1, which can be nuclear, cytoplasmic, or located at the outer mitochondrial membrane, and on the partners of cyclin D1, such as CDK4/6, transcription cofactors, chromatin-modifying enzymes, and cytosolic proteins^4,5^. We previously showed that cyclin D1 controls the unfolded response pathway (UPR) in the endoplasmic reticulum of MM cells^6^. In addition, the transcriptomic profiling of MM cell lines overexpressing a *CCND1* transgene has revealed that cyclin D1 expression may be linked to changes in the transcription of genes involved in metabolism^6^.

A role of cyclin D1 in the control of energy production and metabolism has been documented. Indeed, cyclin D1 acts in association with CDK4 to inhibit mitochondrial respiration by repressing the nuclear respiratory factor 1 (NRF1) transcription factor^7^. Independently of CDK4/6, cyclin D1 binds the voltage-dependent anion channel (VDAC), preventing ADP from reaching the mitochondrial matrix^8^. Cyclin D1 represses peroxisome proliferator-activated receptor α (PPARα), thereby inhibiting fatty acid oxidation^9^. In hepatocytes, cyclin D1 represses gluconeogenesis and oxidative phosphorylation (OxPhos) by inhibiting the PPARγ co-activator, peroxisome proliferator-activated receptor α (PGC1α). This inhibition is CDK4/6-dependent and is also dependent on the fasting and refeeding of hepatocytes^10^.

With the aim of identifying new cellular pathways that could be targeted in MM cells, we investigated the role of cyclin D1 in energy metabolism in this disease. We found that cyclin D1 controlled glycolysis through two concomitant mechanisms involving hexokinase 2 (HK2), the first enzyme in this pathway: (a) by binding to HK2 at the outer mitochondrial membrane, and (b) by acting as a hypoxia-inducible factor-1α (HIF1α) cofactor in the transcription of the *HK2* gene. We found that HK2 overexpression triggered a shift from mitochondrial respiration to oxidative glycolysis. Thus, HK2 appears to be a master regulator of energy metabolism in MM cells, opening up new opportunities for the development of novel treatments.

## Results

### Cyclin D1 expression leads to the Warburg effect in MM cells

**Fig. 1:**
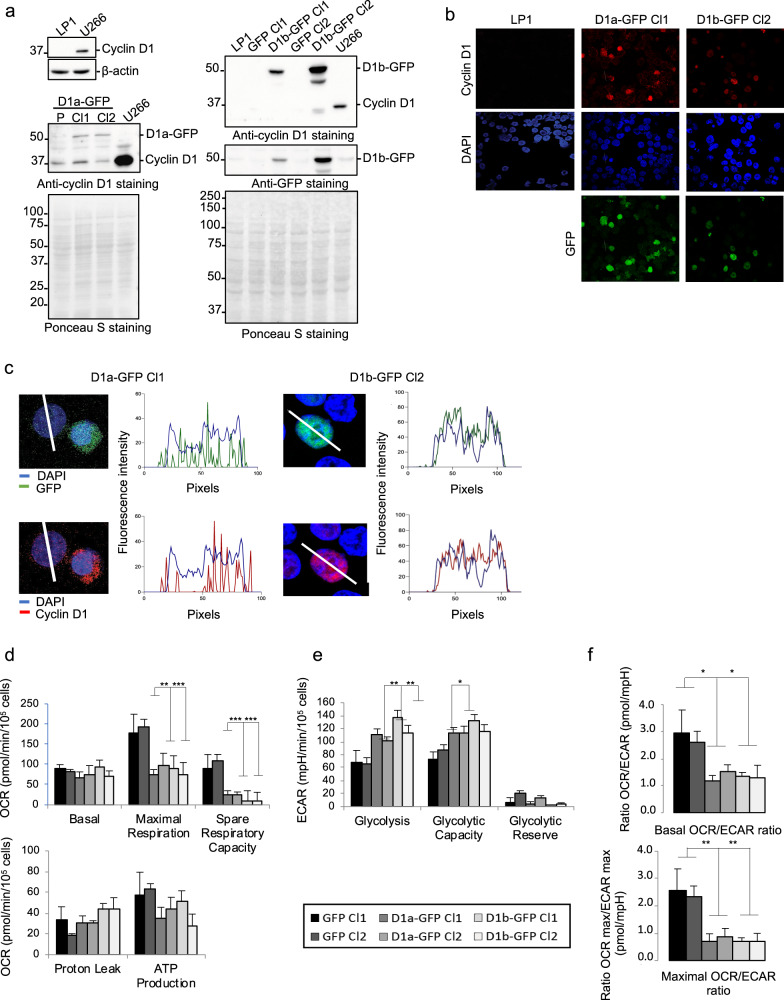
Long and short forms of cyclin D1 induce a metabolic shift in LP1 cells. **a** Whole-cell extracts were obtained from cultured LP1, D1a–GFP Cl1/Cl2, D1b–GFP Cl1/2, and U266 MM cells. Proteins were subjected to SDS-PAGE, transferred onto nitrocellulose sheets that were stained with Ponceau S, and analyzed by WB. The blots were incubated with the indicated Abs. An anti-β-actin Ab was used as a loading control. The sizes of the molecular weight markers are indicated on the blots. **b** LP1, D1a–GFP Cl1, and D1b–GFP Cl2 cells were analyzed by IF and confocal microscopy after DAPI (in blue) or cyclin D1 (in red) staining, or for GFP (in green) expression (×180 magnification). **c** Merged and enlarged (×3) images of representative cells were processed with the ImageJ software, and the curves of fluorescence intensity (FI, in AU) as a function of distance (in pixels) along the white line crossing one cell were exported. **d**–**f** The evaluation of mitochondrial respiration and glycolytic function in LP1 GFP (Cl1 and Cl2, in black and dark gray), D1a–GFP (Cl1 and Cl2, in medium gray), and D1b–GFP-expressing cells (Cl1 and Cl2, in light gray and white) was performed on a Seahorse analyzer. Basal and maximal respiration, spare respiratory capacity, ATP-linked, and proton leakage (**d**) were determined by measuring OCR (pmoles/min/10^5^ cells) as described in Figure S1a,c. Glycolysis, glycolytic capacity, and glycolytic reserve (**e**) were determined by measuring ECAR (mpH/min/10^5^ cells) as described in Fig. S1b, d. Measurements were normalized for cell concentration, and the means ± SEM from four independent experiments are plotted. **f** The basal OCR/ECAR and maximal OCR/ECAR ratios were determined in the same clone. *p < 0.05; **p < 0.01; ***p < 0.001 in paired *t* tests.

We previously showed that the cytoplasmic form of cyclin D1 downregulates mitochondrial respiration in mature B cells^8^. As a means of discriminating between the cytoplasmic and nuclear functions of cyclin D1, we genetically modified the parental LP1 MM cell line, which does not produce endogenous cyclin D1, and selected stable clones (Fig. 1a). LP1-derived clones expressed either only the green fluorescent protein (GFP), as a control, or one of the cyclin D1-GFP fusion proteins: the canonical long form (D1a) or a short cyclin D1 form (D1b) deleted from the last 21 amino acids, including Tyr286, a phosphorylation site essential for nuclear export and proteasome degradation^11^. Hereafter, we refer to the LP1 clones (Cl) as GFP, D1a–GFP, or D1b–GFP. Recombinant protein production was verified by western blotting (WB), and the subcellular distribution of the proteins was determined by indirect immunofluorescence (IF) (Fig. 1a, b). The D1a isoform was both nuclear and cytoplasmic, as previously reported^6^, whereas the isoform D1b was strictly nuclear in D1b–GFP cells (Fig. 1c).

We investigated the possibility of a Warburg effect driven by cyclin D1, by assessing both the respiratory and glycolytic metabolism of the LP1 clones through determinations of cellular oxygen consumption rate (OCR, Fig. 1d, f) and extracellular acidification rate (ECAR, Fig. 1e, f). Maximal respiration rates were much lower in clones (Cl1 and 2) expressing D1a–GFP (73.6 and 97.8 pmoles/min/10^5^ cells, respectively) and D1b–GFP (91.8 and 75.7 pmoles/min/10^5^ cells, respectively) than in the two GFP-control clones (179.6 and 192.5 pmoles/min/10^5^ cells, respectively). This decrease in maximal respiration rate was linked to a mean 75% (D1a Cl1/2 cells) or 89% (D1b Cl1/2 cells) decrease in respiratory spare capacity (Fig. 1d), characterized by a lack of response to the uncoupler 2,4-dinitrophenol (DNP) (Fig. S1a). It was accompanied by a tendency toward an increase in proton leakage from the mitochondrial membrane and a decrease in mitochondrial ATP production. The modification of respiratory metabolism in the presence of cyclin D1a or D1b was accompanied by increases in both glycolysis (+56% in D1a–GFP and +85% in D1b–GFP) and glycolytic capacity (+42% in D1a–GFP and +55% in D1b–GFP) (Fig. 1e, Fig. S1b). These results suggest that both forms of cyclin D1 promote the Warburg effect. Further evidence to support this hypothesis is provided by the decrease in both OCR/ECAR (−54% in D1a–GFP and −50% in D1b–GFP) and OCR_max_/ECAR_max_ (−67% in D1a–GFP and −71% in D1b–GFP) ratios (Fig. 1f). These results reveal a new function of cyclin D1 in energy production in MM tumor cells. Moreover, both cyclin D1 isoforms (D1a or D1b) had an impact on metabolism.

### Cytoplasmic cyclin D1 and HK2 are colocalized at the outer mitochondrial membrane

**Fig. 2:**
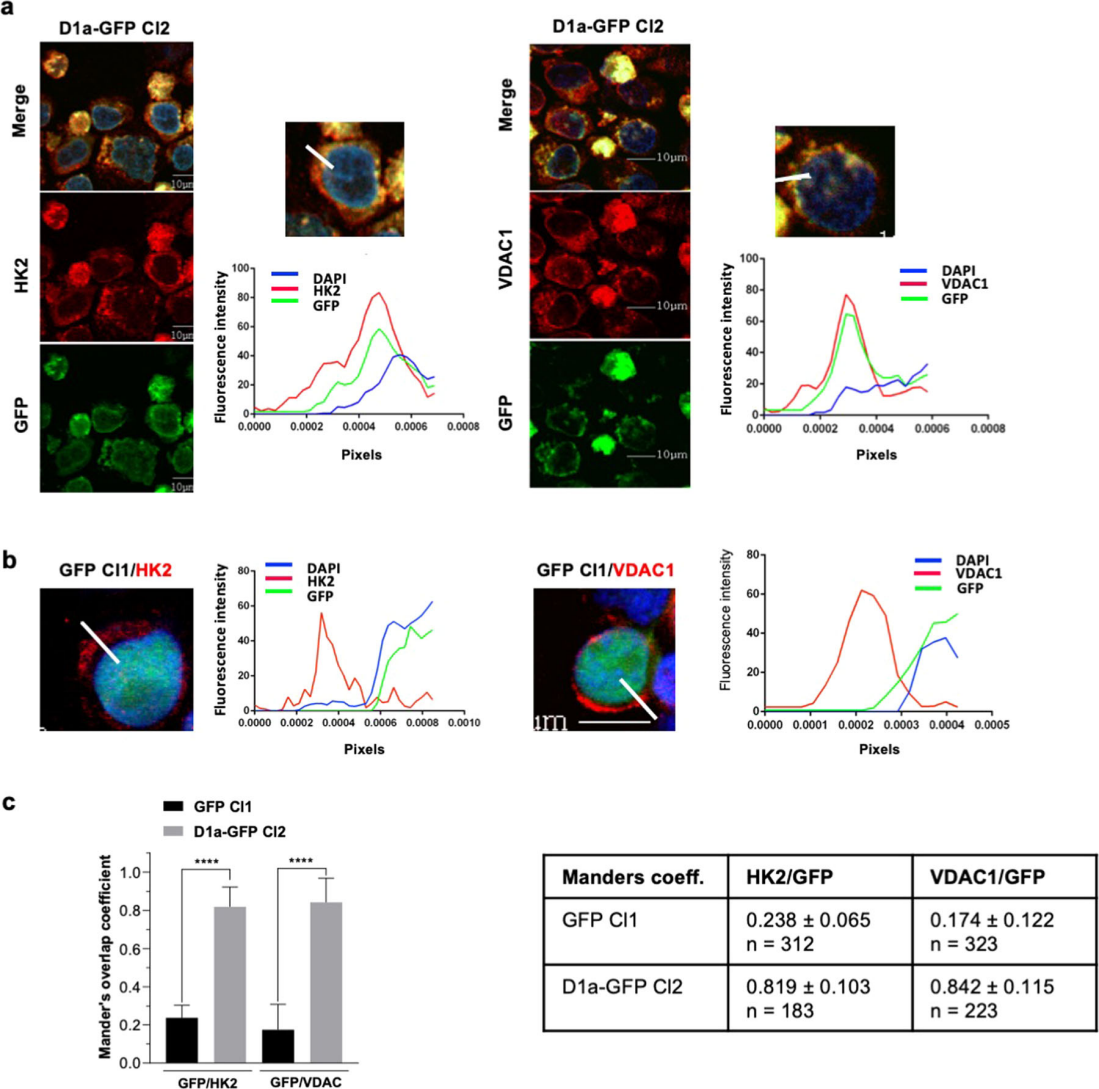
Cyclin D1 and HK2 are bound in D1a–GFP-expressing LP1 cells. **a** Cultured GFP-D1a Cl1 cells were cytospun and analyzed by confocal microscopy for GFP expression (in green) or after DAPI (in blue), HK2, or VDAC (in red) staining (×180 magnification). Representative images are shown. In the merged and enlarged (×3) image, the FI of each fluorophore was estimated with ImageJ, and data were exported to generate the curves of fluorescence intensity as a function of the distance. This experiment was performed twice. **b** The localizations of VDAC1 and HK2 and GFP fluorescence were studied in LP1–GFP cells and exported with ImageJ as previously described. **c** The colocalization of VDAC1 and HK2 with cyclin D1a was confirmed with the Manders’ overlap coefficient from stained cells on three independent images for each staining condition (https://imagej.nih.gov/ij/). Means ± SD are indicated on the histograms and in the accompanying table, together with the number of cells analyzed for each condition. ****p < 0.0001 with the *t* test.

In B cells, cyclin D1 decreases mitochondrial respiration by competing with HK2 at the outer mitochondrial membrane for VDAC binding, ATP reduction, and metabolite supply^8^. We investigated the possible regulatory function of cyclin D1a on the OCR in MM cells, by analyzing the subcellular distribution of cyclin D1. Using a combination of GFP expression and staining for HK2 or VDAC1, we performed IF studies on LP1 cells expressing cyclin D1a (Fig. 2a). As expected, cyclin D1a colocalized with HK2 and VDAC1, whereas the GFP control did not (Fig. 2b). The calculated Manders’ overlap coefficient was greater than 0.8, confirming the colocalization of cyclin D1a with HK2 and with VDAC1 (Fig. 2c). Consistent with previous results, mitochondrial cyclin D1 probably bound HK2, suggesting that it may couple glycolysis and OxPhos in MM cells.

### Nuclear cyclin D1 controls oxidative glycolysis independently of CDK4/6

**Fig. 3:**
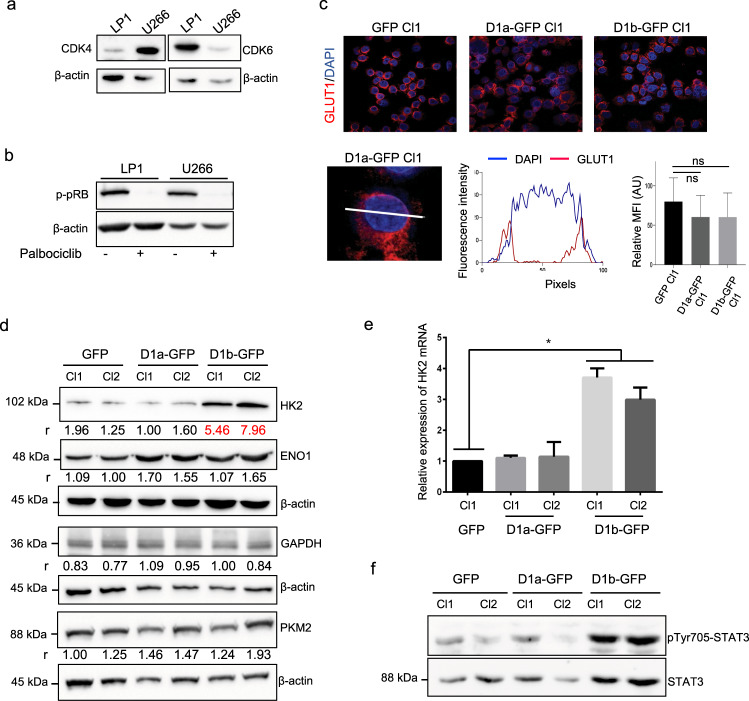
Nuclear cyclin D1 increases HK2 mRNA and protein levels. **a** Whole-cell proteins were obtained from the LP1 and U266 MM cell lines, subjected to SDS-PAGE, transferred onto nitrocellulose sheets, and incubated with the indicated Abs. As before, an anti-β-actin Ab was used as a loading control. **b** LP1 cells were treated with 2 μM palbociclib for 24 h (+) or with vehicle (0.01% DMSO) and harvested. Whole-cell proteins were prepared and analyzed by WB with the indicated Abs. **c** LP1-derived clones were cytospun on glass slides, stained with an anti-GLUT1 primary Ab and a goat Alexa Fluor 546-conjugated anti-rabbit IgG as a secondary Ab (in red), and counterstained with DAPI (in blue). The slides were analyzed by confocal microscopy (×180 magnification). In the merged and enlarged image (×3), the FI of each fluorophore was estimated with ImageJ software and data were exported. The FI was recorded from 90 individual cells from each clone analyzed, with ImageJ software. The means and SD of fluorescence intensity (MFI) are presented in the histograms. ns, not significant in the *t* test. **d** Whole-cell proteins were obtained from GFP and D1a/b-GFP-expressing clones, subjected to SDS-PAGE, and transferred onto nitrocellulose sheets. Blots were cut into strips and incubated with the indicated Abs. An anti-β-actin Ab was used as a loading control. The level of each glycolytic enzyme was estimated by densitometry and normalized against the β-actin level. The calculated ratios (*r*) are indicated under the blots. **e** RT-qPCR analysis of *HK2* transcripts in LP1-derived clones. The results are presented as the fold change in cyclin D1-expressing cells (2^−ΔΔCt^ values) relative to GFP-expressing cells (normalized to 1). The experiments were performed twice, with triplicate samples. The data shown are means ± SD, *p < 0.05 in the *t* test. **f** Whole-cell proteins were purified from cultured clones and analyzed by WB with the indicated Abs as previously described.

Cyclin D1, associated with CDK4, controls glucose metabolism in hepatic carcinoma cells^12^. We therefore investigated whether CDK4 and/or CDK6 modulated the Warburg effect. We first confirmed that both these kinases were expressed in MM cells (Fig. 3a). We then treated cyclin D1b-expressing cells with palbociclib at a dose that inhibited CDK4/6 kinase activity and dephosphorylated pRB (Fig. 3b). The impact of the pharmacological inhibition of CDK4/6 on metabolism was evaluated by measuring the OCR and ECAR, together with proton leakage and ATP production. No significant difference was observed between treated and untreated cells (Fig. S2). These data indicate that the effects of cyclin D1b on metabolism may involve a mechanism independent of CDK4/6-associated kinase activity.

### Nuclear cyclin D1 expression is associated with an increased HK2 level

The enhanced glycolysis capacity of cyclin D1-expressing cells may result from higher levels of glucose uptake or of the production and activity of glycolytic enzymes. We therefore performed IF experiments to analyze the levels of GLUT1, the only glucose transporter expressed in MM cells^13^ (Fig. 3c), and WB to assess the levels of four key glycolytic enzymes (Fig. 3d). The GLUT1, ENO1, GAPDH, and PKM2 proteins were detected in D1a–GFP-expressing clones, at levels similar to those in the GFP controls. By contrast, HK2 levels were much higher in D1b–GFP-expressing cells than in cells expressing D1a–GFP or GFP (Fig. 3d). The increase in HK2 abundance was accounted for by changes in transcription, as demonstrated by the 3.8- and 3.0-times higher *HK2* mRNA levels in D1b–GFP clones (Cl1 and 2) than in GFP and D1a–GFP clones (Fig. 3e).

The signal transducer and activator of transcription 3 (STAT3) pathway has been shown to control metabolic reprogramming toward oxidative glycolysis in MM cells^14^. We therefore also assessed STAT3 production and activation. We found that the amount of Tyr705-phosphorylated STAT3 increased with cyclin D1b levels. This increase was partly explained by an increase in total protein levels (Fig. 3f).

These data revealed that both the nuclear and cytoplasmic forms of cyclin D1 target HK2, through different mechanisms, in MM cells.

### Cyclin D1 and HIF1α are bound within the nucleus

**Fig. 4:**
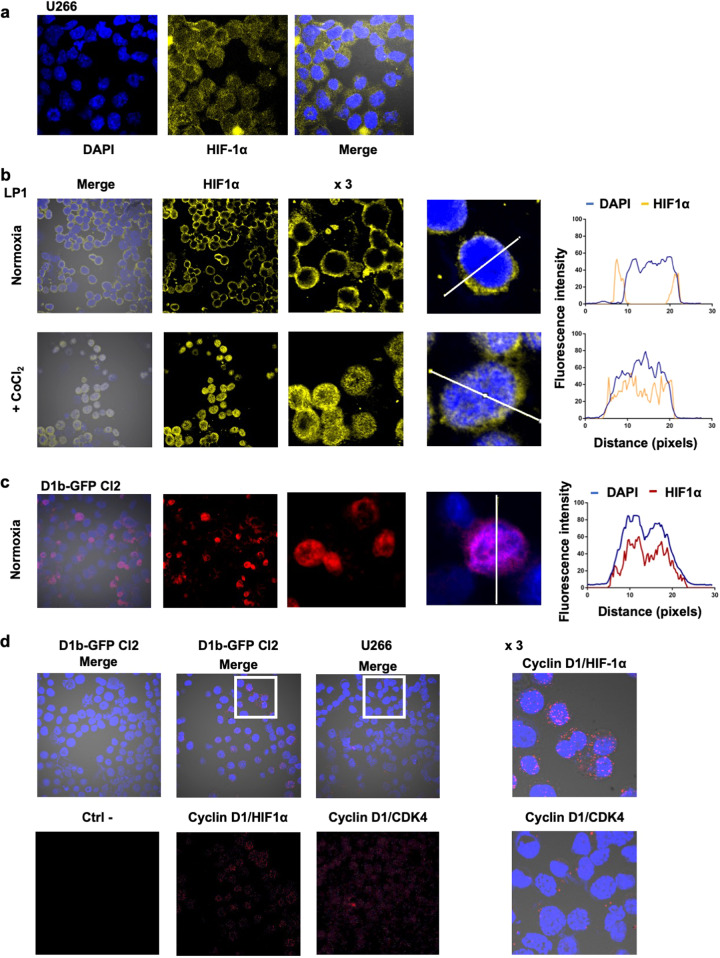
The nuclear form of cyclin D1 binds HIF1α. **a** U266 cells were cultured under normoxia and analyzed by IF and confocal microscopy after DAPI (in blue) or HIF1α (in yellow) staining (×180 magnification). **b** LP1 cells were cultured under normoxia or treated with 300 μM CoCl_2_ overnight and analyzed after DAPI (in blue) or HIF1α (in red) staining. Merged images of representative cells were processed with ImageJ software, and the curves of FI as a function of distance were exported. **c** D1b–GFP Cl2 cells were cultured under normoxia and analyzed by IF, as previously described. **d** Duolink PLA technology was used on D1b–GFP Cl2 cells treated with CoCl_2_ (to enhance the signal) and U266 cells (as a control), to investigate cyclin D1/HIF1α and cyclin D1/CDK4 interactions, respectively. Slides were incubated with the primary Abs, except for the negative control (Ctrl−), and then with the secondary Abs conjugated to the MINUS and PLUS probes. The slides were counterstained with DAPI before confocal microscopy examination (×180 magnification). Representative fields (white squares) were enlarged (×3).

In tumor cells, including MM cells, activated STAT3 upregulates HIF1α, a master transcriptional regulator of hypoxia^14^. In the B-cell lineage, hypoxia induces changes in metabolism, including, in particular, glycolysis, through HIF1α^15^. HIF1α is constitutively expressed in primary MM cells and MM cell lines^16^. Consistent with these data, HIF1α was stabilized in U266 and LP1 cells under normoxia, but was found strictly in the cytoplasm (Fig. 4a, b, Fig. S3a). Following the treatment of LP1 cells with CoCl_2_, which mimics hypoxia, HIF1α was mostly nuclear (Fig. 4b). Remarkably, under normoxia, HIF1α was cytoplasmic in D1b–GFP Cl2 cells, but was also found in the nucleus in some cells (Fig. 4c). Moreover, in those cells, HIF1α and cyclin D1b were colocalized in the nucleus (Fig. S3b). The activation of HIF1α in hypoxic conditions was confirmed, in LP1 and LP1-derived cells, by the induction of one of its targets, BCL2-interacting protein 3 (BNIP3) (Fig. S3c). By contrast, the expression of another target of HIF2α, the octamer-binding transcription factor 4 (OCT4), was not affected.

As cyclin D1b and HIF1α were colocalized, we examined their interaction in situ, in the proximity ligation assay (PLA). No red fluorescent signal was detected in the negative control, whereas, in the positive control (U266 cell line expressing both cyclin D1 and CDK4, Fig. 3a, b), interactions between cyclin D1 and CDK4 were visible (red dots) in both the cytoplasmic and nuclear compartments (Fig. 4d). Cyclin D1 interacted with HIF1α in the nucleus of the D1b–GFP Cl2 clone. The number of red dots, each corresponding to a single-cyclin D1/HIF1α complex, counted in 100 independent cells, ranged from 1 to 49 (mean: 14 ± 10.4) per cell. These data indicate that cyclin D1 binds HIF1α in the nucleus of MM cells, and suggest a possible role in regulating its transcriptional activity.

### Cyclin D1 and HIF1α cooperate at the proximal promoter of *HK2* to control the expression of this gene

**Fig. 5:**
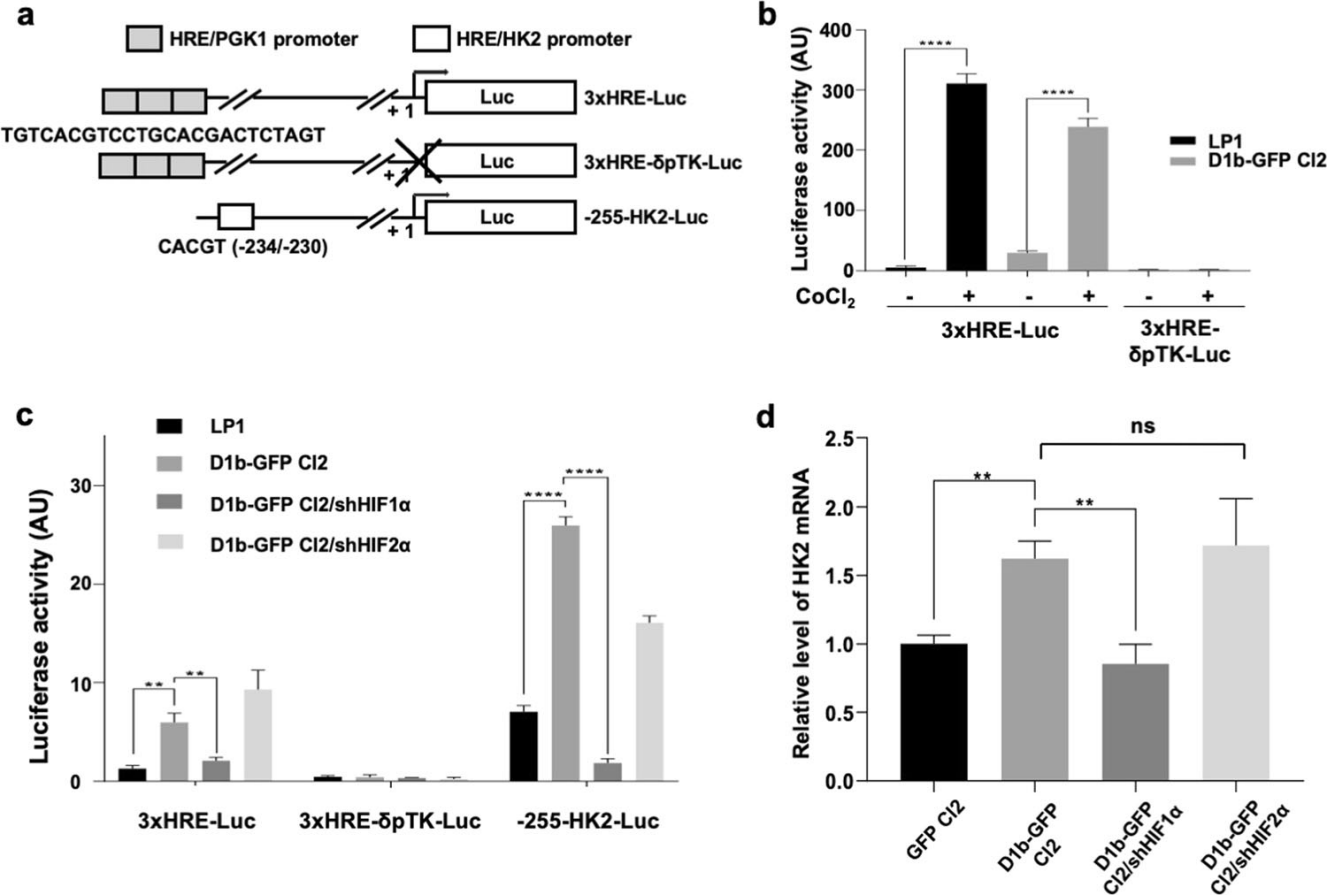
A cyclin D1–HIF1α axis regulates *HK2* transcription. **a** Schematic representation of the luciferase reporter plasmids. **b** LP1 and D1b–GFP Cl2 cells were treated for 6 h with 300 μM CoCl_2_ to mimic hypoxia or left untreated (−), then transfected by electroporation with either the 3× HRE-Luc or 3× HRE-δpTK-Luc plasmids. Luciferase activity was measured 48 h later, and data were normalized according to the total protein content of the samples. The experiment was performed twice, with triplicate samples. Data are means ± SD. ****p < 0.0001 in the *t* test. **c** LP1, D1b–GFP Cl2, and D1b–GFP Cl2 cells transduced with lentiviruses bearing shRNAs against HIF1α or HIF2α were electroporated with 3× HRE-Luc, 3× HRE-δpTK-Luc, or −255-HK2-Luc reporter plasmids. Luciferase activity was measured and data were normalized. The experiments were performed twice, with triplicate samples. *p < 0.05, ***p < 0.001, ****p < 0.0001 in the *t* test. **d** RT-qPCR analyses of *HK2* transcripts in GFP Cl2 and D1b–GFP Cl2 cells uninfected or infected with shHIF1α or shHIF2α lentiviruses. The results are presented as the fold change in D1b–GFP Cl2 cells (2^−ΔΔCt^ values) relative to GFP Cl2 cells (normalized to 1). **p < 0.01, ns not significant in the *t* test.

Transcription of the *HK2* gene is controlled by various transcription factors, including HIF1α^17,18^. The hypoxia-response element (HRE), located in the −234/−230 position, has been shown to be the key minimal regulatory region of the *HK2* promoter^19^. We investigated the possible role of cyclin D1b in regulating *HK2* expression by stabilizing HIF1α, in luciferase reporter assays with three expression plasmids (3× HRE-Luc, 3× HRE-pδTK-Luc, and −255-HK2-Luc) (Fig. 5a). Constructs were first validated in LP1 parental and D1b–GFP-expressing cells, by treatment with CoCl_2_ (Fig. 5b). Luciferase activity levels in D1b–GFP Cl2 cells were 4.6 times higher than in LP1 cells transfected with the 3× HRE-Luc plasmid, and 3.7 times higher than in LP1 cells transfected with the −255-HK2-Luc plasmid (Fig. 5c). No luciferase activity was detected when the two cell lines were transfected with the 3× HRE-δpTK-Luc plasmid with a deletion of the proximal promoter, demonstrating the absence of background expression. We demonstrated that the luciferase activity was truly linked to HIF1α, by transducing GFP-D1b Cl2 cells with a shHIF1α-expressing lentivirus. In cells in which HIF1α expression was knocked down, luciferase activity levels were lower by a factor of 2.8 for 3× HRE-Luc and 14.0 for −255-HK2-Luc. The data obtained with the shHIF2α lentivirus construct were ambiguous, because luciferase activity decreased or increased, depending on the reporter plasmid used (−255-HK2-Luc vs. 3× HRE-Luc). Thus, *HK2* expression was enhanced by cyclin D1b, through HIF1α rather than HIF2α, by binding to the HRE minimal binding site within the *HK2* promoter, under normoxic conditions.

For further validation of the involvement of HIF, we then silenced HIF1α or HIF2α in D1b–GFP Cl2 cells and quantified the level of *HK2* expression. *HK2* expression decreased only in HIF1α-silenced cells, by a factor of 1.9 (Fig. 5d). Overall, our data show that cyclin D1 expression activates a HIF1α/HK2 axis, which reprograms cells to increase aerobic glycolysis.

### HK2 is overexpressed in MM cells, and high levels of HK2 are correlated with poor disease-free survival and overall survival

**Fig. 6:**
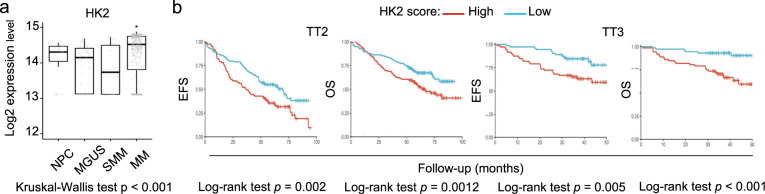
*HK2* is overexpressed in MM patients and is associated with a poor prognosis. **a** Boxplots of *HK2* expression in NPCs (*n* = 22), MGUS (*n* = 40), SMM (*n* = 12), and MM (*n* = 388) samples. A pairwise statistical comparison between groups was performed with a nonparametric Wilcoxon test followed by Benjamini–Hochberg correction (*p < 0.05). **b** Kaplan–Meier curves showing the correlation of *HK2* expression with event-free survival (EFS) and overall survival (OS) in two independent MM cohorts: TT2 (*n* = 243) and TT3 (*n* = 145). High and low scores, defined as above and below the median level of expression, respectively. Log-rank test *p* values are indicated on the curves.

We evaluated the clinical relevance of HK2 expression in MM, by investigating whether the level of *HK2* expression varied with disease progression in publicly available microarray datasets^20,21^. We found that the level of HK2 expression in active MM disease was significantly higher than that in normal plasma cells (NPCs) and premalignant cells, including monoclonal gammopathy of undetermined significance (MGUS) and smoldering MM (SMM) (Fig. 6a). We also analyzed the correlation between *HK2* expression and the survival outcomes of MM patients from two separate clinical trials: TT2 (refs. ^20,21^) and TT3 (ref. ^22^). MM patients with high levels of *HK2* expression had a shorter event-free survival (EFS) and overall survival (OS) than those with low levels of *HK2* expression, in both trials (Fig. 6b). These findings thus suggest that *HK2* may be involved in disease progression, and that *HK2* expression could potentially be used as a marker for the stratification of MM patients.

## Discussion

The metabolism of tumor cells is reprogrammed to maximize the consumption of glucose, to provide a source of carbon for the generation of the nucleotides, amino acids, and lipids required for tumor growth and proliferation. The regulation of metabolism has already been reported as one of the functions of cyclin D1 in oncogenesis^9,12^. We describe here a new mechanism of glucose metabolism regulation in MM cells. The cyclin D1 oncoprotein sustains a Warburg effect through two concomitant, complementary mechanisms: (a) the mitochondrion-associated form of cyclin D1 decreases mitochondrial respiration and, (b) the nuclear cyclin D1 associates with HIF1α to control *HK2* transcription. The resulting increase in *HK2* gene transcription leads to an increase in HK2 protein levels and, probably, glycolytic activity.

HK2 catalyzes the first rate-limiting glycolytic reaction: the conversion of glucose into glucose-6-phosphate (G6P), which then passes through the glycolysis and pentose phosphate pathways. HK2 has been shown to cause a shift from OxPhos to glycolytic metabolism^17^. This shift occurs when normal cells are exposed to hypoxia, and in tumor cells under normoxia. HK2 is found mostly at the outer mitochondrial membrane. Its binding to VDAC1 promotes catabolic glucose metabolism and protects cells against apoptosis^23^. In FLT3/ITD leukemic cells, the glycolytic phenotype is supported by a substantial upregulation of HK2 binding to VDAC at the outer mitochondrial membrane^24^. The shift of mitochondrial respiration to glycolysis triggered by the direct channeling of ATP through VDAC1/HK2 complexes is also supported by our previous data^8^. We show here that, as in other cancer cells, HK2 is located mostly at the outer mitochondrial membrane, consistent with a role in energy modulation in MM cells.

Both the cytoplasmic and nuclear forms of cyclin D1 regulate metabolism, through various mechanisms^8^, but we report here a previously unknown function of cyclin D1 as a cofactor of HIF1α for the regulation of *HK2* gene transcription. Cyclin D1 is a well-known regulator of gene transcription, upregulating the expression of some genes and downregulating that of others^25^. Cyclin D1 modifies chromatin structure at active promoters. It associates with coactivators displaying histone acetyltransferase (HAT) activity and modulates this HAT function. Cyclin D1 thus modifies the histone acetylation of local chromatin at transcription factor-binding sites, repressing or inducing gene transcription. It also regulates methyltransferase activity, activating protein arginine methyltransferase (PRMT) 5 and its cofactor, MEP50, in particular^26^. The overproduced PRMT5 cooperates with cyclin D1 to drive mouse lymphomagenesis^27^, highlighting the major role of cyclin D1-mediated transcription in lymphoid tumorigenesis.

Cyclin D1 has no known DNA-binding sequence, but its abundance is correlated with transcriptional activity^25^, confirming its role as a cofactor. We report here a new function of cyclin D1 as a cofactor for HIF1α, highlighting two original points: (1) cyclin D1 acts independently of CDK4/6, and (2) cyclin D1 activates *HK2* transcription. Indeed, all the transcription factors (DMP1, FOXO, STAT3, and RUNX3) and nuclear receptors (androgen receptor, PPAR, and thyroid receptor) regulated by cyclin D1, with the exception of the estrogen receptor, repress gene transcription^4^. These observations have implications for the management of MM patients. CDK4/6 inhibitors, which have broad potential for the treatment of cancers, including MM^28^, should counteract the Warburg effect, and HIF1α appears to be a potent target for antimyeloma therapy. The chemical inhibition of HIF1 by echinomycin has been reported to have a cytostatic effect on MM cell lines, and acts in synergy with another drug to induce apoptosis^16^. Further studies are required to determine whether HIF1α is a druggable target.

HK2 overexpression is observed in many solid tumors, and is associated with a poor prognosis^29^. Most MM tumor cells express HK2^30^. Our analysis of two cohorts of MM patients further indicated that high *HK2* levels were correlated with a shorter EFS and OS. Consistent with our results, high levels of *HK2* expression are significantly associated with an aggressive phenotype in patients with primary diffuse large B-cell lymphoma (DLBCL), suggesting that HK2 level could be used to stratify DLBCL patients^31^. In MM patients, HK2 level could be regarded as a prognostic marker, and even as a theranostic marker, independently of molecular subgroup.

Many attempts have been made to inhibit the increase in glycolysis observed in cancer cells, but there is currently no successful treatment based on this approach. Pharmacological agents that target glucose metabolism are generally highly cytotoxic^32^. However, the knockdown of *HK2* expression with an antisense oligonucleotide (HK2-ASO) has a strong cytostatic effect on MM cell lines and in xenograft models^30^. Interestingly, HK2-ASO acts in synergy with an inhibitor of mitochondrial complex I (metformin), both in vitro and in vivo. We have reported that cyclin D1 disturbs the redox status of MM cells by generating reactive oxygen species^33^. Combined therapy with both HK2 inhibitor and ROS scavengers may efficiently induce both cytostasis and cytotoxicity. Alternatively, an HK2 inhibitor could be combined with drugs targeting relevant signaling pathways. HK2 inhibitors associated with proteasome inhibitors, such as bortezomib or carfilzomib, which seems to be more potent, could be assessed. The identification of HK2 partners would also help to define new antimyeloma targets. In this context, VDAC seems to be a promising target, because HK2/VDAC interactions control OxPhos and apoptosis. In conclusion, HK2 that regulates the first step of glycolysis, appears to be a pertinent target for antimyeloma therapy.

## Materials and methods

### Cell culture and infection with lentivirus

The LP1 and U266 MM cell lines were maintained in culture in RPMI 1640 medium (Lonza, Basel, Switzerland) supplemented with 10% fetal calf serum (PAA Laboratories, Toronto, Canada), 2 mM L-glutamine, and antibiotics (Lonza), under a humid atmosphere at 37°C. Cell authentication was based on short tandem repeat (STR) profiling (DSMZ, Braunschweig, Germany). The GFP- and cyclin D1a–GFP-expressing LP1-derived clones have been described elsewhere^6^. For generation of the cyclin D1b–GFP-expressing clones, LP1 cells were stably transfected by electroporation with the cyclin D1b–EGFP-C1 plasmid kindly provided by D. Salomon. Transfected clones were obtained by limiting dilution and maintained under selective pressure with G418 (500 ng/ml, Lonza). GFP expression was checked regularly by flow cytometry. Cells were treated overnight with 300 μM CoCl_2_ (Sigma-Aldrich, Saint-Louis, MO), as previously described^34^, to mimic hypoxia and stabilize HIF1α.

The sequences of shRNAs against HIF1α and HIF2α and their cloning in the bicistronic lentiviral vector TRIP-ΔU3-EF1α have been described elsewhere, together with the control shRNA^35,36^. Concentrated lentiviral supernatants were generated by the LNOx laboratory. LP1 and D1b–GFP Cl2 cells (5 × 10^7^/100 ml) were incubated with concentrated virus at a multiplicity of infection of 4–12 × 10^6^ units/ml for 24 h in complete culture medium. Cells were carefully washed after transduction and amplified for analyses.

### Seahorse assays

OCR and ECAR data were obtained with a Seahorse XF96 Flux analyzer from Seahorse Bioscience (North Billerica, MA, USA). Experiments were performed according to the manufacturer’s instructions. Briefly, LP1 culture medium was replaced with XF BASE MEDIUM supplemented with glutamine (2 mM) and lacking bicarbonate (pH 7.4), and clones were used to seed XF96 cell culture plates at 10^5^ cells/well, with poly-d-lysine. Cells were incubated for 1 h at 37°C without CO_2_, and measurements were performed. Sequential injections of 10 mM glucose, 1 μM oligomycin, 100 μM DNP, and a mixture of 0.5 μM rotenone and antimycin A were performed in accordance with the supplier’s technical specifications, to determine the principal metabolic parameters (Fig. S1).

### Indirect IF and confocal microscopy analysis

MM cells were cytospun on Superfrost glass slides, fixed in 4% paraformaldehyde, and permeabilized in 0.5% Triton X-100. The slides were stained with primary antibodies (Abs, Table S1), and then with Alexa Fluor 488 (green), 546 (orange), or 633- (red) conjugated goat anti-rabbit or mouse IgG and counterstained with DAPI (blue, Molecular Probes, Invitrogen, Carlsbad, CA). Slides were observed with a confocal fluorescence microscope (Fluoview FV 100, Olympus, Rungis, France). The colocalization of VDAC, HK2, and cyclin D1 proteins was investigated with ImageJ software (available at https://imagej.nih.gov/il/), as previously described^37^.

### Proximity ligation assay

PLA technology was used to detect cyclin D1–HIF1α protein interactions in situ. We used the Duolink In Situ Red Starter Kit (DUO92101, Sigma-Aldrich) according to the manufacturer’s instructions, as previously described^37^. Cells were fixed, permeabilized, and incubated with the primary Abs, as described for IF (Table S1). Duolink secondary Abs were then added. These secondary Abs were conjugated to oligonucleotides capable of forming a closed circle by base pairing and ligation in Duolink ligation solution. This situation occurs only when the Abs are in close proximity. As a negative control, no primary Ab was added to the reaction mixture. Slides were observed with a confocal fluorescence microscope (Fluoview FV 100, Olympus).

### Luciferase reporter assays

The 3× HRE-Luc plasmid was provided by Addgene (#26371, Cambridge, MA); it contains three HRE (24-mers) from the *PGK1* (phosphoglycerate kinase 1) gene^38^. The 3× HRE-δpTK-Luc plasmid, deleted of the promoter, served as a control^39^. The −255-HK2-Luc plasmid contains the minimal HRE-binding site of the *HK2* promoter inserted into the pGL3-basic luciferase reporter vector^19^. Cells were transfected with LP1 and LP1-derived clones by electroporation (Gene Pulser Xcell, Bio-Rad, Hercules, CA) with 5 μg of the corresponding plasmids. Luciferase activity was measured 48 h after transfection, with the Bright-Glo Luciferase assay (Promega, Madison, WI) and a VICTOR X4 plate reader (PerkinElmer, Waltham, MA, USA). Data were normalized by total protein, which was used as a proxy for the number of transfected and living cells, and was estimated with the Bradford assay (Bio-Rad).

### Western blotting

Whole-cell protein extracts were prepared from cultured cells with the M-PER Mammalian Protein Extraction Reagent (Pierce Biotechnology, Waltham, MA), according to the manufacturer’s instructions. The methods used for WB have been described elsewhere^40^. Protein levels were estimated by densitometry (ChemiDoc XRS+, ImageLab software, Bio-Rad). The background of each image was subtracted from the bands of interest, and the density of each protein of interest was then normalized against the density of the control housekeeping protein, β-actin (*r*).

### Real-time PCR

Cultured cells were washed with PBS, and cell pellets were used for RNA isolation with TRIzol reagent (Invitrogen), according to the manufacturer’s instructions. RNA samples were subjected to reverse transcription (RT) with the GoScript reverse transcriptase (Promega), as recommended by the supplier. The resulting cDNAs were used for quantitative (q) real-time PCR. PCR primers were designed with the Primer-BLAST and Primer 3 websites (Table S2), and used to amplify cDNAs generated by RT. PCR was performed in GoTaq Master Mix (Promega), according to the supplier’s recommendations. RT-qPCR was performed with a StepOnePlus real-time PCR system (Thermo Fisher Scientific, Waltham, MA) and standard parameters. Both *RPLP0* and *ACTB* were used as housekeeping genes for normalization of the results. Each reaction was conducted in triplicate. Relative levels of *HK2* expression were calculated by the 2^−ΔΔCt^ method.

### MM patient datasets

The MM expression microarray datasets, GSE5900 and GSE2658 (reanalyzed under GSE24080) (refs. ^19,20,41^), were downloaded from the Gene Expression Omnibus (GEO) repository (https://www.ncbi.nlm.nih.gov/geo/). Raw CEL files from the GEO repository were redeposited as GSE24080 with the CEL files included and the annotations updated^41^. Raw CEL files (Affymetrix GeneChip Human Genome U133 Plus 2.0) and sample annotations were acquired from both the GSE5900 and GSE24080 datasets, whereas the GSE2658 dataset was used solely to obtain information relating to MM subtype and myeloid contamination status. We removed 145 of the 637 samples available for download, because of myeloid contamination, as shown by the authors^21^, and another six samples were removed due to incomplete annotation. Furthermore, we retained only arrays for which RNA and hybridization qualities were good, satisfying all the following criteria: scaling factor below 3 and 3′/5′ ratios for β-actin and GAPDH below 3 and 1.25, respectively. We removed another 24 samples after quality-control assessment in R software (version 3.3) with BioConductor’s SimpleAffy package^42^. In total, 462 samples remained for further analysis (Table S3), comprising 22 samples of NPCs, 40 samples of MGUS, 12 samples of SMM, and 388 MM samples from two separate clinical trials: TT2 (*n* = 243) and TT3 (*n* = 145) (refs. ^20–22^), both performed at the University of Arkansas for Medical Sciences. Probe-intensity values were normalized with the gcrma (background adjustment using sequence information) package (Wu and Irizarry, https://rdrr.io/bioc/gcrma/). For the *HK2* gene, the probe with maximum mean expression was considered for the analysis. High and low scores were attributed to MM patients, using median *HK2* expression as the cutoff. Survival analysis was performed in the survminer package of R (https://CRAN.R-project.org/package=survminer).

### Statistical analyses

Student’s *t* test was used to determine the significance of differences between two experimental groups. Data are presented as means ± SD or SEM, and were analyzed in two-tailed tests. The *p* values are indicated as follows: *p < 0.05; **p < 0.01; ***p < 0.001; ****p < 0.0001; ns, not significant.

For transcriptomic data, all statistical analyses were performed in the R statistical environment. Pairwise statistical analyses comparing *HK2* expression in various clinical subtypes with that in NPCs were performed with nonparametric Wilcoxon tests followed by Benjamini–Hochberg correction. Adjusted *p* values less than 0.05 were considered significant. Univariate survival analysis was performed by Kaplan–Meier methods, after setting a threshold based on the median level of gene expression, and comparisons were performed in Mantel–Cox log-rank tests. Survival analyses adjusted for age and sex (multivariate) were performed with Cox proportional hazard model, and Wald’s test was used to assess the significance of calculated hazard ratios for each covariate (Table S4).

## Supplementary informations

Supplementary informations

Supplementary tableS3
