# Supplementary material for: Cyclin D1 targets hexokinase 2 to control aerobic glycolysis in myeloma cells
Source: Oncogenesis. 2020 Jul 24;9(7):68. doi: 10.1038/s41389-020-00253-3 (PMC7381668; doi:10.1038/s41389-020-00253-3)
Supplement: Supplementary file 1 — Supplementary informations [file 41389_2020_253_MOESM1_ESM.docx]

**Cyclin D1 targets hexokinase 2 to control aerobic glycolysis in myeloma cells**

M Caillot^1^, J Bourgeais^2^, H Dakik^3^, É Costé^1,3^, NM Mazure^4^, É Lelièvre^5^, O Coqueret^5^, O Hérault^2,3^, F Mazurier^3^, B Sola^1^

**Supplementary informations**

**Table S1.** Primary antibodies used in the study

| **Target** | **Reference** | **Origin or reference** | **Assay** |
| --- | --- | --- | --- |
| β-actin | #4970 | Cell Signaling Tech. | WB (1/1 000) |
| BNIP3 | ab10433 | Abcam | WB (1/200) |
| CDK4 | sc-260 | Santa Cruz Biotech. | PLA (1/50)  WB (1/200) |
| CDK6 | sc-177 | Santa Cruz Biotech. | WB (1/200) |
| Cyclin D1 | DCS-6  ab40754 | Santa Cruz Biotech.  Abcam | PLA (1/200)  WB (1/1 000) |
| ENO1 | sc-100812 | Santa Cruz Biotech. | WB (1/500) |
| GAPDH | sc-137179 | Santa Cruz Biotech. | WB (1/1 000) |
| GFP | sc-8334 | Santa Cruz Biotech. | WB (1/200) |
| GLUT1 | ab652 | Abcam | IF (1/500) |
| HIF1α | home-made | Richard et al., 1999 | IF (1/500)  PLA (1/500)  WB (1/2 000) |
| HK2 | ab227198  #2106 | Abcam  Cell Signaling Tech. | IF (1/100)  PLA (1/100)  WB (1/1 000) |
| OCT4 | #2750 | Cell Signaling Tech. | WB (1/1 000) |
| PKM2 | ab137852 | Abcam | WB (1/500) |
| pSer807/811-RB | #9308 | Cell Signaling Tech. | WB (1/500) |
| STAT3  pTyr705-STAT3 | #4904  #9131 | Cell Signaling Tech. | WB (1/200)  WB (1/1 000) |
| VDAC1 | ab15895 | Abcam | IF (1/100) |

Peroxidase-conjugated goat anti-rabbit and anti-mouse IgG (H&L) secondary antibodies purchased from Abcam (ab6721 and ab97040, respectively), were used at a dilution of 1/10 000 for WB. Abbreviations : BNIP3, BCL2/adenovirus E1B 19 kDa protein-interacting protein 3; CDK, cyclin-dependent kinase; ENO1, enolase α; GAPDH, glyceraldehyde 3-phosphate dehydrogenase; GFP, green fluorescent protein; GLUT1, glucose transporter 1; HIF, hypoxia-inducible factor; ERK, extracellular signal-regulated kinase; HK2, hexokinase 2; IF, immunofluorescence; PLA, proximity ligation assay; OCT4, octamer-binding transcription factor 4; PKM2; pyruvate kinase M2; STAT, signal transducer and activator of transcription ; VDAC, voltage-dependent anion channel; WB, western blotting.

**Table S2**. Sequence of the primers used in qRT-PCR assays

| Gene | Sequence |
| --- | --- |
| *GAPDH* | F, 5’-CTG ACT TCA ACA GCG ACA CCC-3’  R, 5’-CCC TGT TGC TCT AGC CAA AT-3’ |
| *HK2* | F, 5’-ACT AGA CGA GAG TTT CCT GGT C-3’  R, 5’-CAA CGT CTC TGC CTT CCA CT-3’ |
| *RPLP0* | F, 5’-CCA GGC GTC CTC GTG CAA GTG-3’  R, 5’-TTC CCG CGA AGG GAC ATG CG-3’ |

Abbreviations: F, forward; R, reverse. Primer sequences were designed with the primer 3 software (v4.0, //primer3.ut.ee/).

**Table S3.** List of arrays retained for MM patients analyses (file Table S1.xls)

**Table S4**. Age and sex-adjusted survival analysis of HK2 score in datasets

|  | Coeff. | Exp (Coef) | Se (Coef) | z | *p* |
| --- | --- | --- | --- | --- | --- |
| DFS-TT2 trial (n = 243) | | | | | |
| HK2 | 0.34336182 | **1.409679** | 0.141133549 | 2.432886 | **0.01497902** |
| Age | 0.01160633 | 1.011674 | 0.008365504 | 1.387403 | 0.16531876 |
| Sex (male) | 0.25077927 | 1.285026 | 0.169099817 | 1.483025 | 0.13806775 |
| OS-TT2 trial (n = 243) | | | | | |
| HK2 | 0.29611732 | **1.344628** | 0.16967072 | 1.745247 | **0.08094189** |
| Age | 0.01878024 | 1.018958 | 0.01006102 | 1.866634 | 0.06195275 |
| Sex (male) | 0.24946398 | 1.283337 | 0.20109898 | 1.240503 | 0.21478924 |
| DFS-TT3 trial (n = 145) | | | | | |
| HK2 | 0.68141558 | **1.9766739** | 0.29641928 | 2.2988234 | **0.02151497** |
| Age | 0.02081388 | 1.021032 | 0.01918271 | 1.0850335 | 0.27790682 |
| Sex (male) | -0.28247913 | 0.7539124 | 0.3186482 | -0.8864922 | 0.37535238 |
| DFS-TT3 trial (n = 145) | | | | | |
| HK2 | 1.13120774 | **3.0993975** | 0.42480304 | 2.6628993 | **0.07747058** |
| Age | 0.01820249 | 1.0183692 | 0.02180996 | 0.8345954 | 0.40394556 |
| Sex (male) | -0.41878319 | 0.6578468 | 0.36238889 | -1.1556182 | 0.247837363 |

**Supplementary Figures**

**
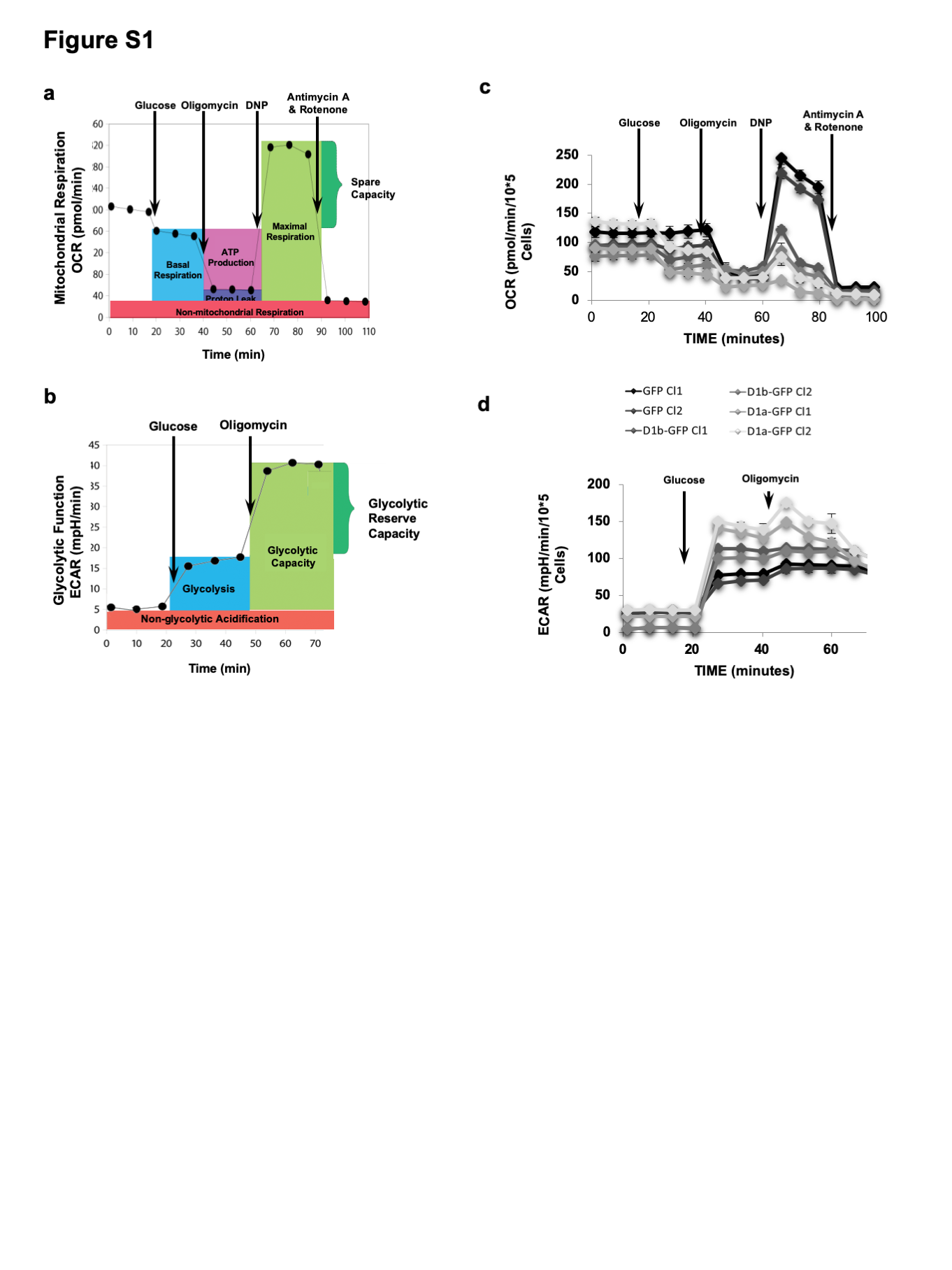
Figure S1. The seahorse assay and experiment timelines.**

**a** Mitochondrial respiration parameters were obtained by the sequential addition of drugs to respiring cells. Baseline OCR was first measured from which basal respiration was deduced by subtracting non-mitochondrial respiration. Oligomycin (1 μM) was then added as an inhibitor of complex V, the resulting OCR was used to derive the ATP production (oligomycin OCR subtracted from basal OCR), and proton leakage (non-mitochondrial respiration subtracted from oligomycin OCR). Next, DNP (100 μM) was then used to collapse the inner membrane gradient to obtain the maximal respiration rate. The maximal respiration capacity was calculated by subtracting non-mitochondrial OCR from DNP OCR. Finally, antimycin A and rotenone (a complex III inhibitor and complex I inhibitor, respectively; 0.5 μM, each) were added to shut down the electron transport chain and to reveal the non-mitochondrial respiration. Reserve (or spare) capacity was calculated by subtracting basal respiration from maximal respiration. **b** Glycolysis parameters of were obtained by the sequential addition of drugs. Baseline ECAR was first measured to determine non-glycolytic acidification. A saturating concentration of glucose (10 mM) was then added as a substrate for glycolysis. The resulting ECAR was used to obtain the rate of glycolysis under basal conditions (by subtracting the non-glycolytic acidification rate from the glucose rate). Oligomycin (1 μM), an inhibitor of complex V, was then used to inhibit mitochondrial ATP production and to shift energy production to glycolysis, revealing the glycolytic capacity (non-glycolytic acidification rate subtracted from oligomycin ECAR). The glycolytic reserve was calculated by subtracting glycolysis from glycolytic capacity. **c, d** Grouped data of original tracings showing changes in OCR (**c**) and ECAR (**d**) in response to the sequential administration (vertical lines) of glucose (10 mM), oligomycin (1 μM), DNP (100 μM), and antimycin A/rotenone (0.5 μM) to LP1 clones expressing cyclin D1a and D1b. Data are expressed as the means of an experimental triplicate ± SEM from a representative experiment. GFP Cl1 and 2 are shown in black and dark gray, D1b-GFP Cl1 and 2 are shown in medium gray, D1a-GFP Cl1 and 2 are in light gray and white.

**
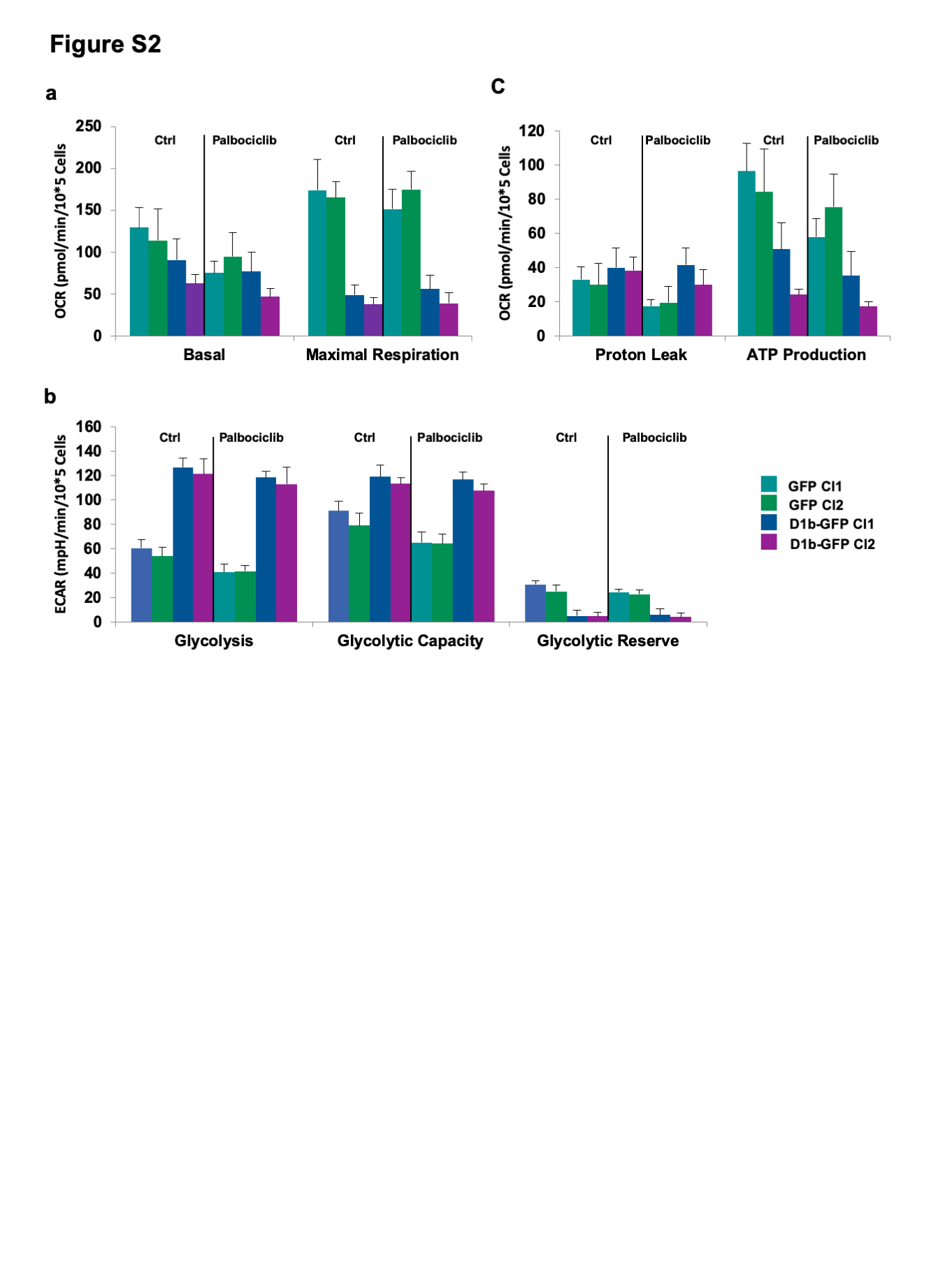
**

**Fig. S2. Palbociclib treatment has no effects on oxygen consumption or lactate production.**

LP1-derived clones (GFP and D1b-GFP) were left untreated or treated with 2 μM palbociclib for 24 h and then assayed with the Seahorse XF96 Flux analyzer according to the experiment timelines described in details in the Figure S1.

**
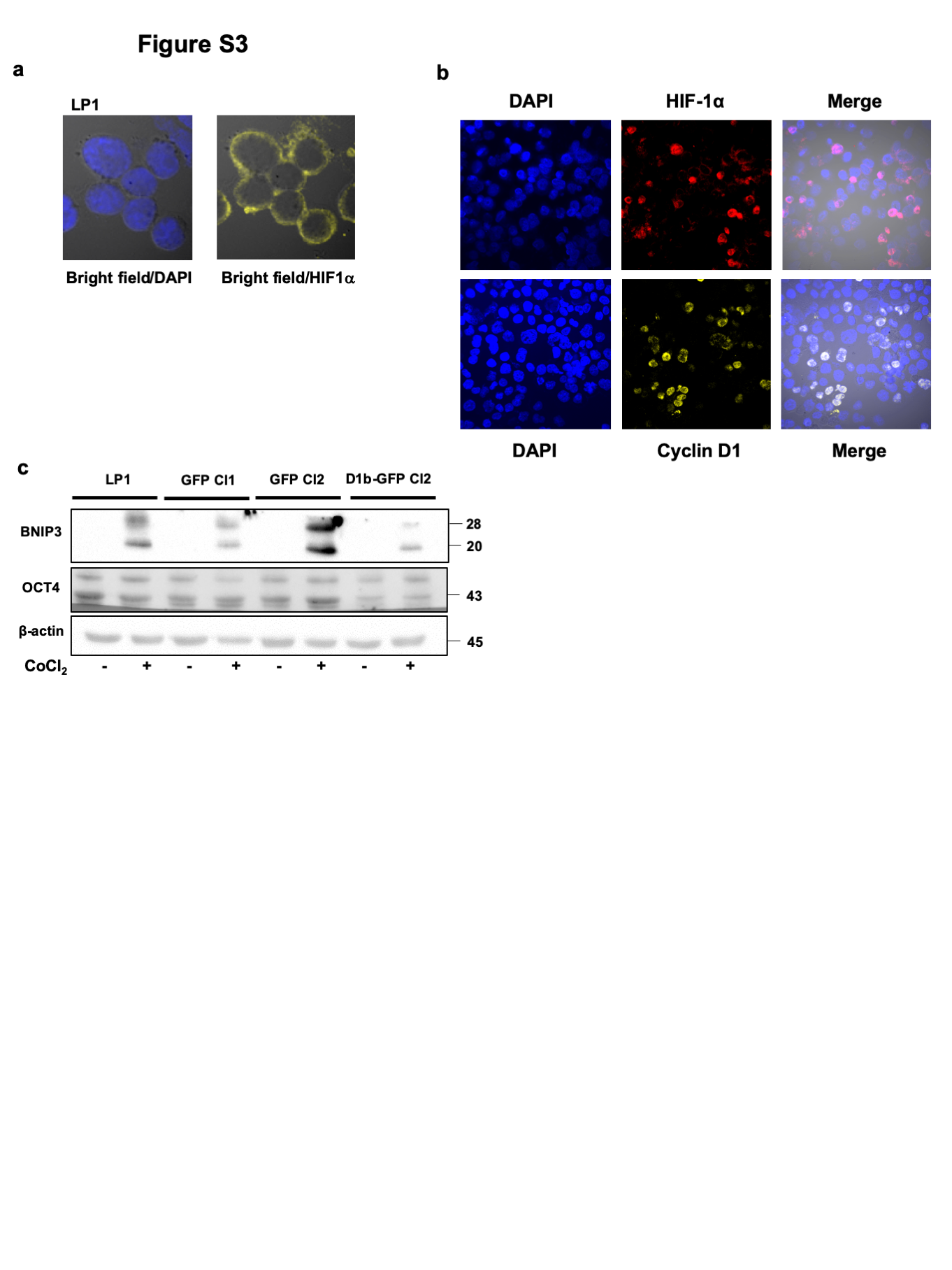
**

**Figure S3**.

**a** LP1 cells cultured under normoxia, were analyzed by IF and confocal microscopy after DAPI (in blue) or HIF-1α (in yellow) staining. Merge and magnified images for bright-field/DAPI and bright-field HIF1α staining. **b** LP1 D1b-GFP Cl2 cells cultured under normoxia were analyzed by IF after DAPI, HIF-1α (in red) or cyclin D1 (in yellow) staining (x180 magnification). **c** Whole-cell proteins extracts were obtained from LP1, GFP- and D1b-GFP-expressing clones cultured under normoxia, treated for 6 h with 300 μM CoCl_2_ (+) or left untreated (-). Proteins were subjected to SDS-PAGE and transferred onto nitrocellulose sheets. Blots were cut into strips and incubated with the indicated Abs. An anti-β-actin Ab was used as the loading control.
